# A user-friendly platform for deep-learning-based image enhancement in scanning X-ray microscopy

**DOI:** 10.1107/S1600577526010076

**Published:** 2026-10-29

**Authors:** Xiaoyin Zheng, Zirui Gao, Ajith Pattammattel, Hanfei Yan, Mingyuan Ge, Yong S. Chu, Yu-chen Karen Chen-Wiegart, Xiaojing Huang

**Affiliations:** a Department of Materials Science and Chemical Engineering Stony Brook University Stony Brook NY11794 USA; b National Synchrotron Light Source II Brookhaven National Laboratory Upton NY11973 USA; University of Malaga, Spain

**Keywords:** scanning X-ray microscopy, deep learning, super resolution, differential phase contrast, X-ray imaging

## Abstract

Images acquired by scanning X-ray microscopy are often limited in spatial resolution by the finite size and profile of the scanning probe. Here, we present a dedicated graphical user interface for physics-informed image enhancement that incorporates the experimentally characterized probe to decouple probe-induced blurring from the measured images. The software integrates experimentally reconstructed probe intensity into the model training and provides an end-to-end workflow for image super-resolution and deblurring. We demonstrate the approach using differential phase-contrast imaging, where the finite scanning probe limits the recovery of fine spatial features. The enhanced images recover spatial details beyond those directly resolved in the measured data. This platform provides a practical and reproducible tool for physics-informed image enhancement during synchrotron X-ray microscopy experiments.

## Abstract 2

A graphical user interface is developed for deep-learning-based image enhancement in scanning X-ray microscopy. By incorporating the intensity of the probe and scan parameters, the graphical user interface enables accessible and reproducible super-resolution and deblurring of scanning X-ray datasets, supporting improved analysis of X-ray fluorescence, differential phase-contrast, and related synchrotron imaging data.

## 1. Introduction

Scanning X-ray microscopy is widely used for multimodal characterization because it provides complementary structural, chemical, and functional information from heterogeneous materials (Chu *et al.*, 2020 ▸; Nazaretski *et al.*, 2017 ▸; Pattammattel *et al.*, 2022 ▸; Yan *et al.*, 2018 ▸). In synchrotron nanoprobe experiments, modalities such as X-ray fluorescence (XRF) and differential phase contrast (DPC) can be acquired simultaneously through raster scanning of a focused X-ray beam across the sample (Quinn *et al.*, 2023 ▸). Acquisition within a common measurement geometry enables direct spatial correlation of complementary contrasts. This is especially important for complex material systems, in which compositional variation, morphology, and local functionality are strongly coupled.

Despite these advantages, the achievable spatial resolution in scanning X-ray microscopy is often limited by the image formation process (Wu *et al.*, 2023 ▸; Huang *et al.*, 2015 ▸). The finite size and shape of the illumination probe introduce spatial blurring, so the recorded signal does not represent an ideal pointwise measurement of the sample. In addition, the scan step size places a sampling limit on the recovered image, and high-throughput acquisition modes such as fly scanning can further reduce image sharpness because signal received by the detector is averaged during continuous sample motion. As a result, fine features may appear broadened, weak boundaries can be obscured, and small-scale variations may be attenuated in the measured data. Reducing these effects experimentally generally requires a smaller probe, finer sampling, or shorter scan increments, creating a practical trade-off among spatial resolution, acquisition time, photon statistics, and radiation dose.

Computational image enhancement has emerged as an attractive strategy for improving the effective resolution of X-ray data without requiring longer acquisition times or increased radiation dose. Among these approaches, deep-learning-based super-resolution and deblurring methods (Nah *et al.*, 2021 ▸; Ren *et al.*, 2020*a* ▸; Abuolaim *et al.*, 2021 ▸; Chen *et al.*, 2022 ▸; Guan *et al.*, 2022 ▸; Ruan *et al.*, 2022 ▸; Zhang *et al.*, 2022 ▸; Xiang *et al.*, 2025 ▸; Zhang *et al.*, 2026 ▸; Ren *et al.*, 2020*b* ▸) have shown strong promise for restoring image details that are degraded by the probe and scanning process. When the degradation mechanism is properly modeled, these methods can recover sharper images while remaining consistent with the experimental measurement conditions. In particular, if the illumination probe and scan parameters are known or can be experimentally characterized, they can be incorporated directly into the restoration workflow to provide more accurate and physically meaningful enhancement.

Despite this promise, applying such methods in practice often requires substantial expertise in data preparation, model selection, training, inference, and result evaluation, which can limit their accessibility during routine beamline experiments. To address this challenge, we developed a graphical user interface (GUI) for probe-informed deep-learning enhancement of X-ray datasets. The platform integrates preprocessing, model selection, training and fine-tuning, inference, and visualization into a unified workflow, allowing non-expert users to directly incorporate experimentally reconstructed probes and scan parameters into the enhancement process. In this way, the GUI provides an accessible and reproducible tool for practical resolution enhancement during synchrotron experiments.

## 2. Software structure

The primary goal of this work is to provide general users with a practical framework for applying deep-learning-based enhancement to scanning-blurred images. In a typical multimodal imaging workflow, users may obtain experimentally reconstructed probes from ptychography, along with phase and amplitude images of the same sample. These complementary data contain information about both the probe and the sample structure, making it possible to integrate them into a unified image enhancement strategy. The proposed framework is intended for images degraded by probe convolution and scan-related blur and is therefore applicable to scanning-based measurements more broadly rather than to a single imaging modality. The deep-learning models are implemented in *PyTorch* and can run on either Linux or Windows systems with at least 16 GB of RAM. The results shown in the paper were obtained using an NVIDIA RTX A6000 GPU with 48 GB of memory and took 16 h for 800 epochs.

**Figure 1:**
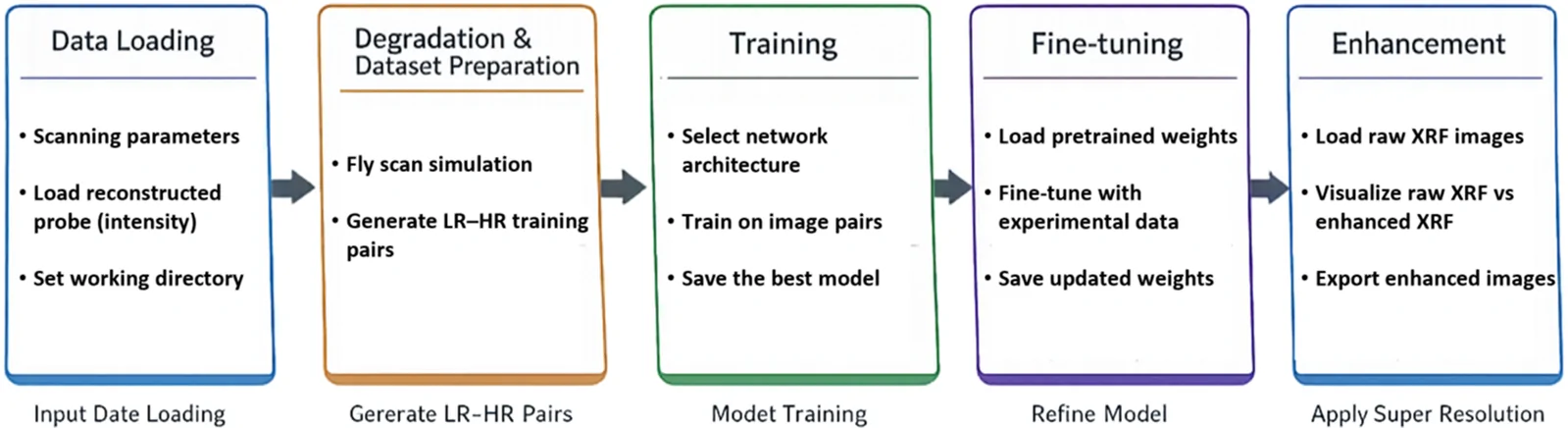
Workflow of the GUI.

The workflow, as shown in Fig. 1 ▸, is as follows: (i) load the probe and scanning parameters into the GUI; (ii) simulate the high- and low-image pairs; (iii) train the chosen deep-learning model with a natural image dataset; (iv) fine-tune the model with the phase-contrast images; (v) enhance the target blurred data.

## 3. GUI design and user interaction

### 3.1. Preprocessing and training dataset

**Figure 2:**
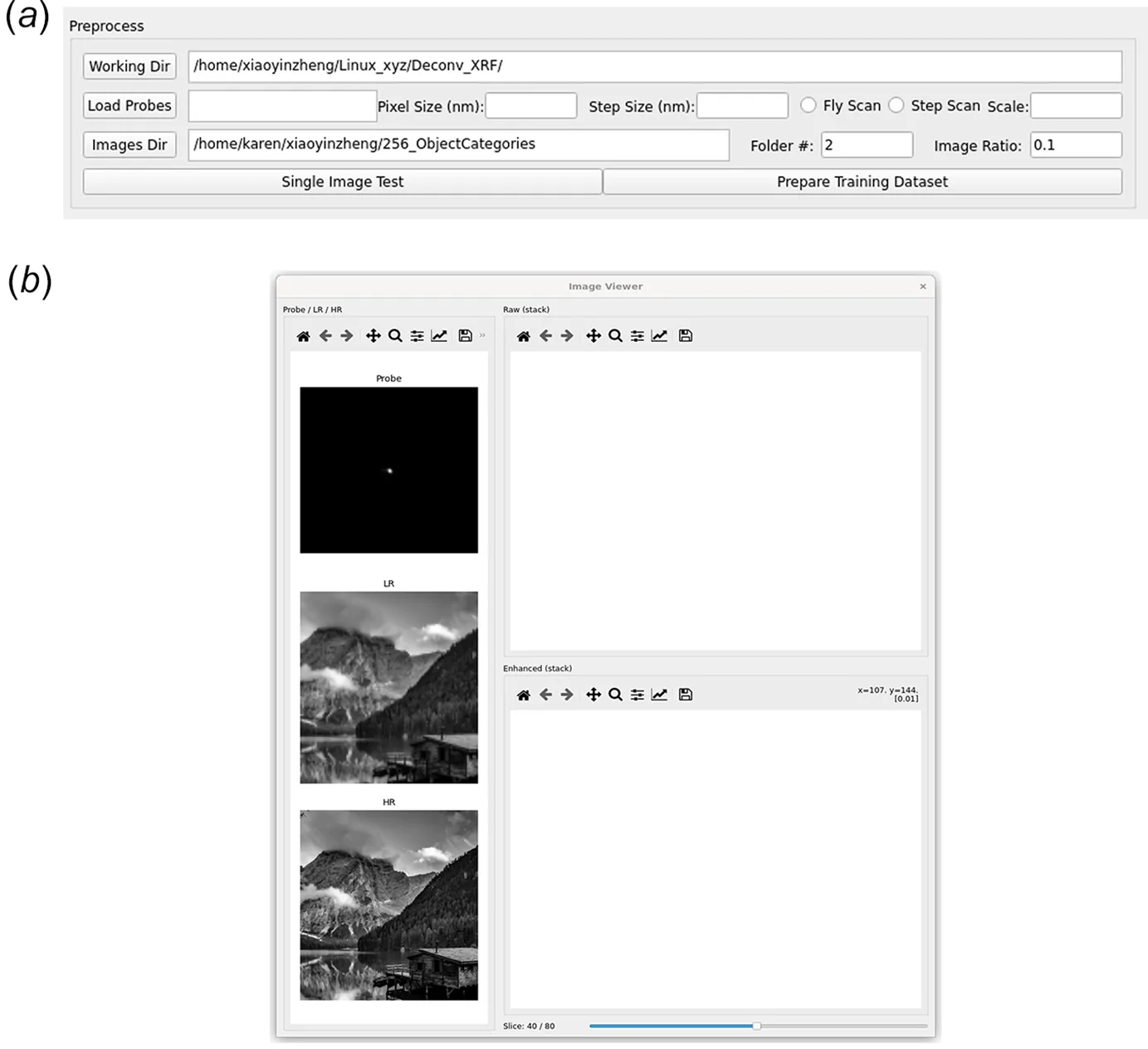
Layout of the preprocessing panel. (*a*) Input panel for selecting working directory, loading probe and specifying experimental parameters. (*b*) Visualization window showing the reconstructed probe, the HR reference image and simulated LR image on the left. The right panel is reserved to display raw and enhanced experimental data.

The GUI provides a dedicated preprocessing module to configure the experimental and imaging parameters required for deblurring XRF images, and to generate paired low-resolution (LR) and high-resolution (HR) images for supervised training. The layout is shown in Fig. 2 ▸. The probe is obtained from ptychography reconstruction with the same experimental condition as the XRF measurement.

The probe profile is loaded through the GUI and displayed in the visualization panel for inspection. The probe intensity is used, and it is consistent with the fact that the measured image data can be modeled as a convolution between the object and the illumination intensity profile.

The scanning step size is taken directly from the experimental setup. The pixel size of the reconstructed probe is determined by the cropped size of the diffraction pattern used in phase retrieval. The real-space pixel size is given by
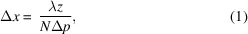
where λ is the X-ray wavelength, *z* is the sample-to-detector distance, Δ*p* is the detector pixel size, and *N* is the number of detector pixels retained after cropping. A smaller cropped diffraction pattern therefore leads to a larger real-space pixel size.

In the GUI, the probe pixel size is read from the reconstructed probe, while the scanning step size is specified by the user from the experimental configuration. The super-resolution upsampling factor is then determined by the ratio between the scanning step size and the probe pixel size.

The GUI allows the user to select between step-scan and fly-scan acquisition modes. In fly-scan mode, the sample is translated continuously while the detector integrates the fluorescence signal over a finite dwell time. As a result, the recorded image data correspond to a moving average of the convolution between the sample and the probe intensity profile, which introduces additional motion-induced blurring along the scanning direction.

This blur is explicitly modeled in the preprocessing stage by convolving the high-resolution image with the probe intensity and integrating the response over the scanning trajectory defined by the scanning step size and fly-scan settings. This forward model is used consistently for both single-image testing and training data generation.

To inspect the effect of the selected preprocessing parameters, the GUI provides an optional single-image simulation using a natural image. An HR reference image is degraded using the probe-aware forward model with the selected scanning parameters to generate a synthetic LR image. The original HR image and the simulated LR image are displayed alongside the probe, allowing users to inspect the modeled blurring before preparing the training dataset. No network enhancement is performed on these reference images during this optional step. They remain visible in the shared visualization window when experimental data are subsequently enhanced.

After the experimental parameters are confirmed, the GUI automatically prepares paired training data by generating LR and HR image pairs. HR images are taken either from natural image datasets or from experimental reference data, while the corresponding LR images are generated using the probe-aware forward model that incorporates the illumination profile, scanning step size, and fly-scan integration. These paired datasets are then exported for subsequent network training and fine-tuning within the same software framework.

This preprocessing workflow ensures that the training data are physically consistent with the data acquisition process and enables seamless integration of experimental probe information into the deep-learning-based super-resolution and deblurring pipeline.

### 3.2. Model selection

The GUI integrates three deep-learning models that target specific enhancement tasks for DPC, XRF and nano-XANES data, enabling users to select an appropriate model according to the imaging modality and data quality.

The first model is based on a residual dense network (RDN) (Zhang *et al.*, 2018*a* ▸) originally developed for probe-aware super-resolution of XRF image data by Wu *et al.* (2023 ▸). This model learns a direct mapping from LR XRF images to HR images by exploiting dense feature reuse and global residual learning, and is trained using paired low- and high-resolution images generated from a physically motivated forward model that incorporates the measured probe intensity. A key advantage of this model is its strong generalization capability: once trained, it can be applied to experimental XRF data acquired with the same probe and scanning configuration without additional fine-tuning, and has been demonstrated to achieve large upsampling factors (up to sevenfold in each lateral direction).

The second model combines a residual channel attention network (RCAN) (Zhang *et al.*, 2018*b* ▸) with a multi-scale residual network (MSRN) (Li *et al.*, 2018 ▸). In this architecture, the RCAN blocks emphasize informative feature channels through channel-wise attention, while the MSRN branch extracts structural information at multiple spatial scales. This design enables efficient feature extraction and improved sensitivity to both fine structures and extended features. The model is particularly suitable for super-resolution and deblurring tasks when strong scale variations are present in the sample morphology.

The third model replaces the multi-scale residual branch with a nonlinear-activation-free network (NAFNet) to further improve robustness to noise (Chen *et al.*, 2022 ▸). By removing conventional activation functions and simplifying the nonlinear transformations, this architecture is optimized for image restoration tasks dominated by noise and blur. In our implementation, this model is mainly used for nano-XANES datasets, where individual XRF frames collected at different energies often exhibit strongly varying noise levels. The RCAN–NAFNet model is able to consistently denoise and deblur XANES frames across the full energy stack, while preserving spatial structures and spectral consistency.

Through this unified model-selection interface, the GUI enables flexible switching between super-resolution-oriented and restoration-oriented networks, facilitating practical deployment of physics-informed deep-learning models.

### 3.3. Training and fine-tuning panel

After the training datasets are prepared in the preprocessing module, users can directly load the paired LR and HR image stacks through the training tab of the GUI. The LR images are used as network inputs, while the corresponding HR images serve as ground-truth labels. Both datasets are stored and loaded as multi-image tensors, enabling efficient batch processing during training.

**Figure 3:**
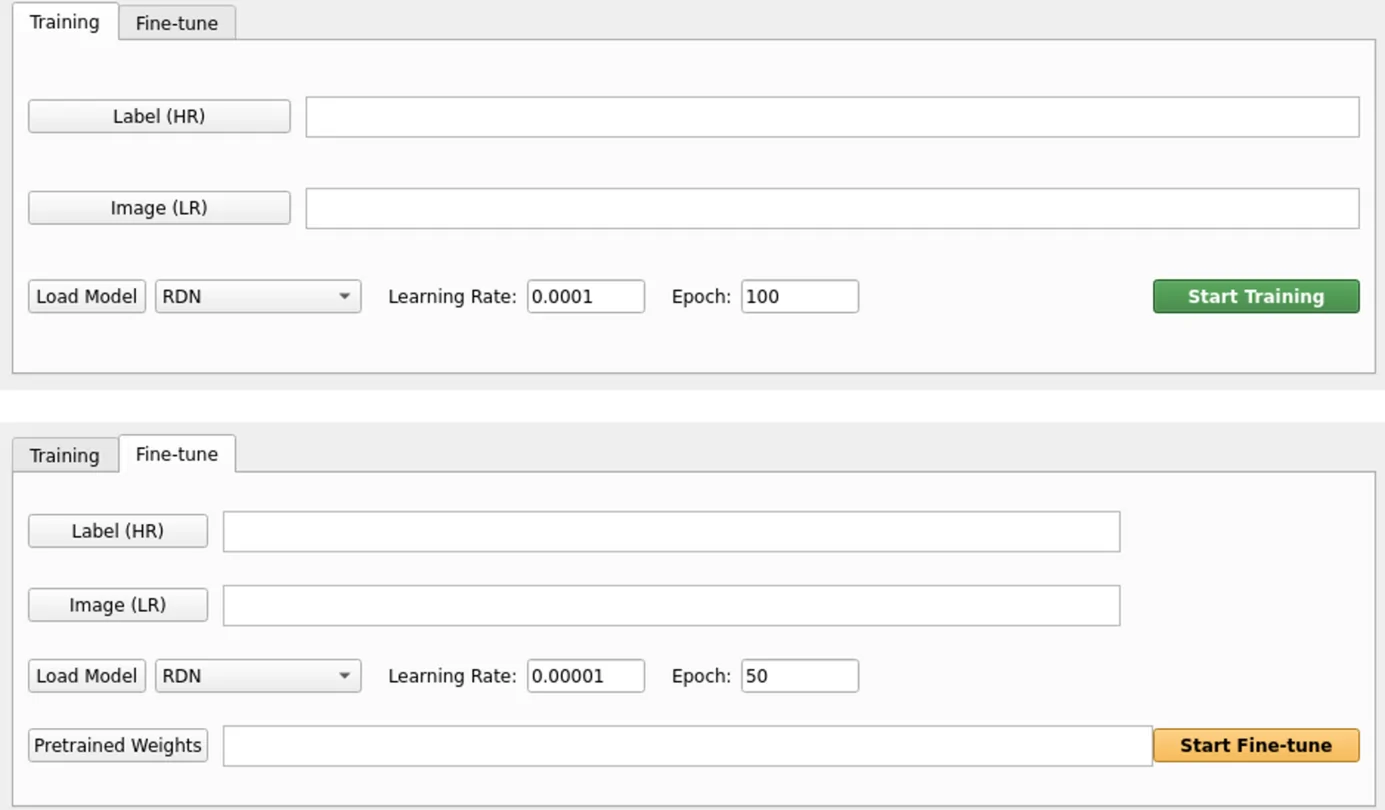
User interface for model training and fine-tuning. The training panel (top) enables loading of HR labels and LR input images, selection of the deep-learning architecture, and specification of training hyperparameters, including learning rate and epoch number. The fine-tuning panel (bottom) includes the same core settings, with the addition of pretrained weight loading for continued optimization of a previously trained model.

The training interface, shown in Fig. 3 ▸, allows users to select one of the integrated network architectures and to configure the main optimization parameters, including learning rate and number of training epochs. Two working modes are supported: training a model from scratch and fine-tuning an existing pretrained model. In the fine-tuning mode, previously trained weights can be loaded and further optimized using new datasets acquired under different probes, scanning conditions, or noise levels.

During training, progress information and status messages are displayed in the GUI in real time. This integrated design enables users to perform dataset loading, model selection, training, and fine-tuning within a single graphical environment, facilitating rapid adaptation of deep-learning models to different XRF and nano-XANES experiments without the need for external training scripts.

### 3.4. Enhancement and visualization of X-ray images

**Figure 4:**
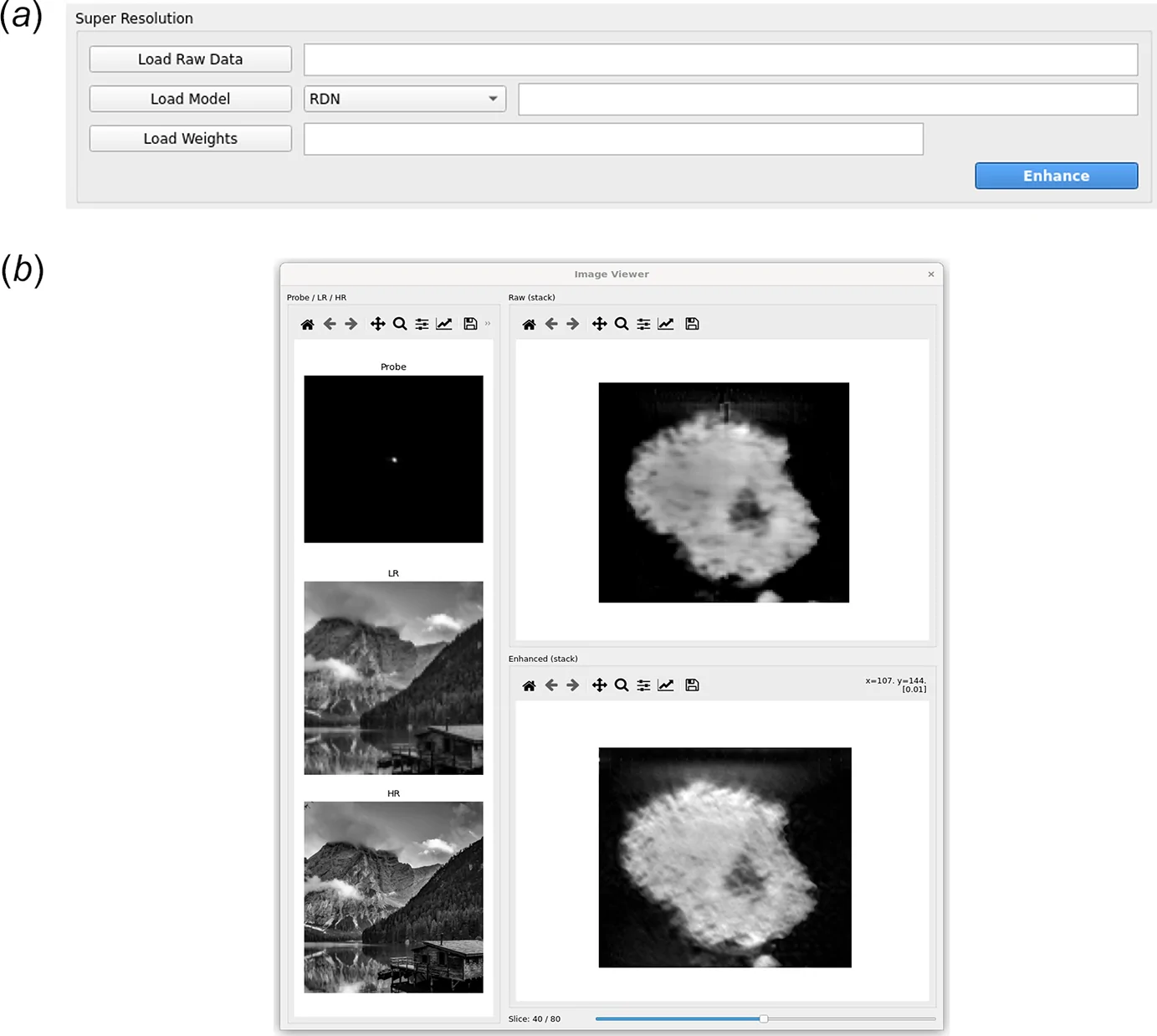
Overview of the GUI for X-ray image enhancement, illustrated using DPC data. (*a*) The super-resolution panel provides controls for raw data import, model selection, trained weight loading, and execution of the enhancement. (*b*) The shared visualization window displays the probe and the natural-image LR–HR reference pair retained from the optional preprocessing simulation on the left, and the raw and enhanced DPC images of an NMC cathode particle on the right. The reference pair illustrates the simulated degradation and is not generated during inference on the experimental data. The slice slider appears only for image-stack inputs and allows browsing through individual two-dimensional frames.

The enhancement module provides an interactive interface for applying the selected deep-learning model to experimental XRF, DPC, and nano-XANES data. The layout is shown in Fig. 4 ▸. The experimental example in Fig. 4 ▸(*b*) shows raw and enhanced DPC images of a lithium nickel manganese cobalt oxide (NMC) secondary particle. This concentration-gradient cathode material has an average composition of LiNi_0.6_Mn_0.2_Co_0.2_O_2_ and was prepared from a concentration-gradient hydroxide precursor (Wu *et al.*, 2023 ▸). Users can first load the raw image or image stack, select a trained network architecture, and specify the corresponding trained model weights. The GUI then performs probe-aware inference using the parameters defined in the preprocessing stage, including the scanning mode and upsampling factor.

Both single images and multi-frame stacks (*e.g.* energy-resolved nano-XANES datasets) are supported. For stack data, the enhancement is applied frame by frame using the same network and parameters, ensuring consistent restoration across the entire energy series. This is particularly important for nano-XANES measurements, where spectral analysis requires spatially and spectrally consistent image quality.

The raw and enhanced experimental images are displayed in the same visualization window for comparison. An interactive slice slider is shown only when the input contains a stack of two-dimensional images. This functionality depends on the input structure rather than the imaging modality: it supports nano-XANES energy stacks and XRF or DPC datasets supplied as image stacks, while single-image inputs are displayed without the slider. The stack index identifies an individual frame and does not necessarily represent a spatial slice through a reconstructed volume. The probe and any natural-image reference pair retained from preprocessing remain visible to document the simulated degradation; they are not ground-truth references for the experimental sample.

After enhancement, the restored images can be exported directly from the GUI for subsequent quantitative analysis, such as nano-XANES spectral fitting or correlative multimodal studies. This integrated enhancement and visualization workflow enables fast feedback during beam time and allows users to immediately assess the impact of different models and parameters on experimental data quality.

## 4. Application – enhancement of DPC data

We demonstrate the application of the proposed GUI to a multi-model dataset acquired from an NMC secondary particle (Wu *et al.*, 2023 ▸). To distinguish from previously reported XRF results and show the generalized applicability of the method, here we show the enhancement on the DPC imaging channel. DPC can be obtained using a scanning X-ray microscope by analyzing the transmitted far-field diffraction pattern, where the specimen induces a slight shift of the forward beam that is proportional to the local gradient of the sample-induced phase, as ∂ϕ/∂*x*,*y* = Δ_*x*,*y*_Δ2π/(λ*z*), where ∂ϕ/∂*x*,*y* are the phase gradients in the horizontal and vertical directions, Δ_*x*,*y*_ are the shifts of the diffraction pattern, Δ is the detector pixel size, and *z* is the sample-to-detector distance. The integrated phase can then be estimated by
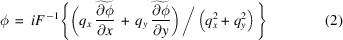
(de Jonge *et al.*, 2008 ▸; Menzel *et al.*, 2010 ▸). In contrast to ptychography, which reconstructs the full complex transmission function of the sample through iterative phase retrieval, DPC directly provides a phase-gradient image from the forward transmission without reconstructing the wavefield. At the HXN beamline, where DPC is typically acquired in fly scan mode, the signal at each pixel is integrated along the continuous scan direction, so that the measured DPC map represents a blurred version of the phase-gradient. The resolution is thus limited by the probe size and scanning step size. Therefore, when the probe profile is known from ptychography reconstruction, the DPC data can be naturally enhanced using the supervised deep-learning method.

We collected a DPC dataset from an NMC secondary particle derived from a concentration-gradient hydroxide precursor with an average composition of Ni_0.6_Mn_0.2_Co_0.2_O_2_ (Wu *et al.*, 2023 ▸). The hard X-ray nanoprobe was focused by a Fresnel zone plate (FZP) with an outermost zone width of 30 nm. A total of 80 two-dimensional projections were collected evenly distributed from 0° to 180° with a dimension of 105 × 120 pixels and pixel size 66.7 nm. Based on the scan step relative to the target pixel size, we apply an upsampling factor of seven, yielding an enhanced DPC image of 735 × 840 pixels with a pixel size of 10 nm. After tomographic reconstruction, the raw 3D DPC volume has a size of 120 × 105 × 120 voxels with a voxel size of 66.7 nm, while the enhanced volume has a size of 840 × 735 × 840 voxels with a voxel size of 10 nm.

**Figure 5:**
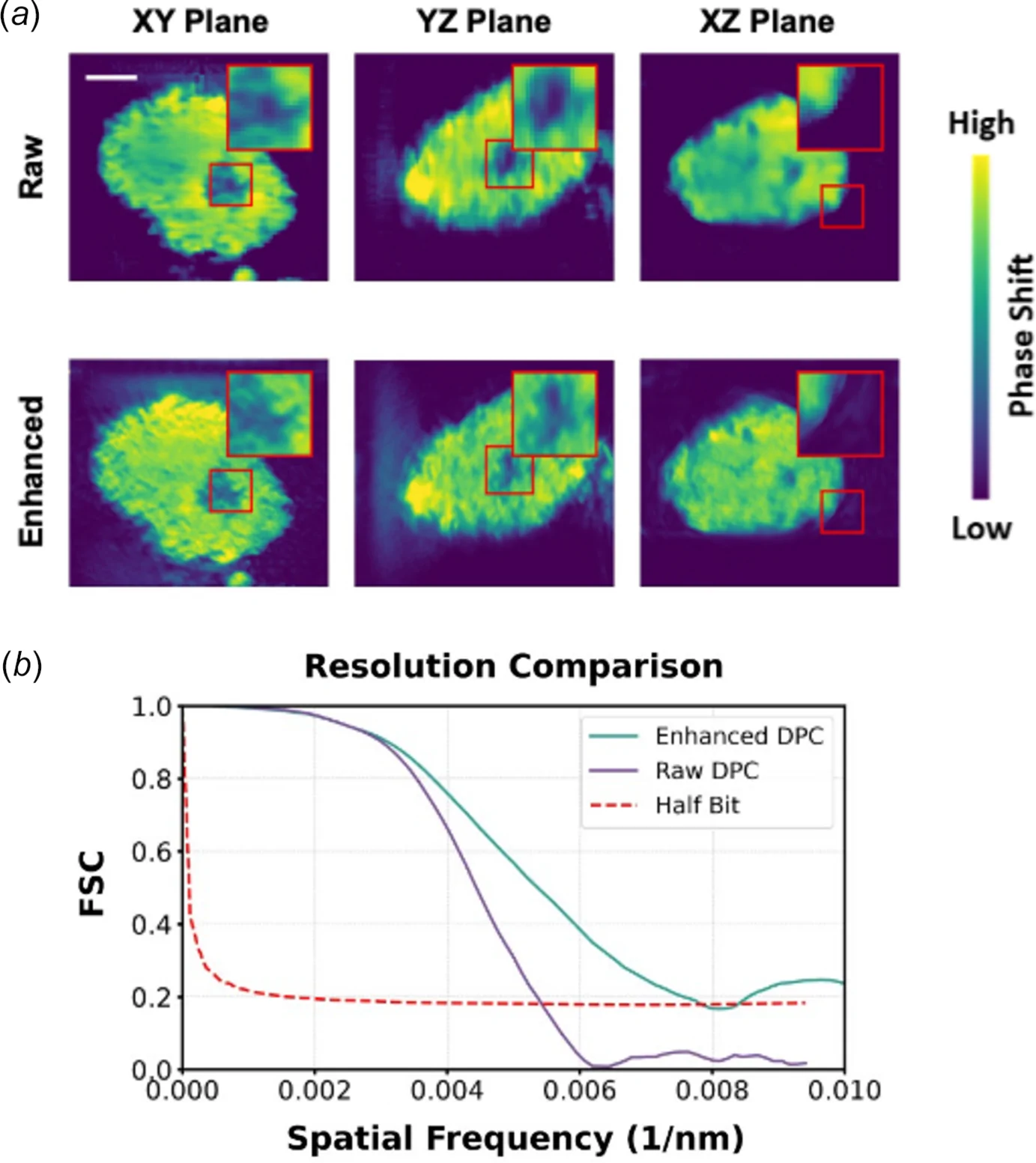
Comparison of the raw and enhanced 3D DPC reconstructions. (*a*) Orthogonal *XY*, *YZ*, and *XZ* slices for the raw (top) and enhanced (bottom) reconstructions, with magnified insets marked by red boxes. Scale bar: 2 µm. (*b*) Fourier shell correlation (FSC) curves and the half-bit threshold; the intersections give resolutions of 186 nm for the raw data and 128 nm for the enhanced data.

Using the GUI, the raw DPC image is loaded, and the enhancement was carried out for each projection. The tomography reconstruction is performed with the ordered subset penalized maximum-likelihood algorithm. It should be noted that the current enhancement is applied only to the lateral dimensions of each 2D projection, while the number and angular sampling of the projections remain unchanged. Therefore, after lateral super-resolution, angular sampling can become an additional resolution-limiting factor in the 3D tomographic reconstruction. The 10 nm voxel size of the enhanced reconstruction represents the sampling interval of the reconstructed volume and does not necessarily correspond to an isotropic spatial resolution of 10 nm. In our previous work (Zheng *et al.*, 2024 ▸), we further addressed this limitation by increasing the angular sampling through reprojection, demonstrating that improved angular sampling can further improve the resolution of the tomography reconstruction. Compared with LR reconstruction, the HR reconstruction maintains the global morphology of the particle while resolving substantially finer structural information in all orthogonal planes [see Fig. 5 ▸(*a*)]. The HR slices exhibit sharper phase contrast, clearer interfaces, and enhanced visibility of fine internal features, whereas the LR slices are characterized by blurred boundaries and reduced local detail. These differences are particularly apparent in the enlarged regions, where the HR reconstruction better resolves local heterogeneity and small-scale contrast variations that are less distinguishable in the LR result. Although the LR reconstruction captures the overall particle shape, its limited spatial definition reduces the interpretability of subtle microstructural features. These results confirm that the HR reconstruction provides a more faithful and informative visualization of the particle interior. To quantify the spatial resolution, we divided the original projections into odd- and even-numbered half-datasets, reconstructed each half separately using the same algorithm, and calculated the Fourier shell correlation (van Heel & Schatz, 2005 ▸) between the two volumes. The resolution was taken as the reciprocal of the spatial frequency at the intersection with the half-bit threshold, yielding 186 nm for the raw reconstruction and 128 nm for the enhanced reconstruction.

The resolution enhancement achieved for DPC here, from 186 to 128 nm, is more modest than the fourfold improvement reported for XRF in our previous work (Zheng *et al.*, 2024 ▸). This difference may partly reflect the greater sensitivity of DPC to noise: extracting small shifts in the transmitted diffraction pattern can be affected by measurement noise, limiting the reliable recovery of fine spatial features.

Following the DPC demonstration, it is also important to consider the practical limitations of the proposed enhancement framework. The method assumes that the reconstructed probe used for training reasonably represents the actual illumination during the experiment. In practice, the probe may vary because of beamline drift, optic alignment, thermal changes, sample position relative to focus, or uncertainties in the ptychography reconstruction. If the actual probe differs substantially from the probe used to generate the LR–HR training pairs, the forward model becomes less representative of the experiment and the enhancement may not represent the true sample feature. In addition, the achievable resolution remains limited by the spatial information retained in the measured data. Spatial frequencies that are strongly suppressed by the probe or insufficiently sampled by the scanning cannot be uniquely recovered, and the reconstruction at those cases may rely more on the learned image prior than on direct measurement. This highlights the distinction between recovering attenuated information and inferring features that were not sufficiently measured. Data quality can also become a limiting factor when the signal is very weak and the measurement is dominated by noise, as can occur in pre-edge frames of nano-XANES datasets. In such cases, the model may not perform reliably on extremely low-count data with significant noise unless it is properly fine-tuned using data with similar signal and noise characteristics.

## 5. Conclusion

In this work, we developed a probe-informed GUI for deep-learning-based enhancement of scanning-blurred X-ray microscopy images. The underlying approach for XRF super-resolution was established in our prior work (Zheng *et al.*, 2024 ▸). Building on that foundation, the GUI translates the method into a practical and user-accessible workflow for routine use. In addition to XRF, we further demonstrate here that the same framework can be extended to DPC data. By incorporating probe intensity and scan parameters into the restoration workflow, the GUI enables users to generate LR/HR training pairs, train or fine-tune deep networks, and perform reproducible super-resolution and deblurring within a single end-to-end workflow. By integrating preprocessing, model selection, inference, visualization, and export into a user-friendly environment, the GUI lowers the barrier for general users to apply deep-learning-based enhancement during synchrotron experiments.

Overall, the proposed GUI provides a practical and extensible tool for improving the effective spatial resolution of scanning-blurred images and enabling higher-quality downstream analysis. Future work will focus on expanding model support, improving robustness across different probe and scan conditions, and further integrating the framework with multimodal imaging workflows.

## Data Availability

The GUI for super resolution is available on GitHub at https://github.com/zxy18301/Super-Resolution-GUI.git.
